# L´association de cellules mésenchymateuses stromales au substitut osseux pour le traitement des pseudarthroses des os longs, une alternative aux greffes spongieuses autologues: à propos d´un cas

**DOI:** 10.11604/pamj.2020.37.234.22809

**Published:** 2020-11-13

**Authors:** Aniss Chagou, Hamza Benameur, Jalal Hassoun, Jaafar Abdeloihab

**Affiliations:** 1 Mohammed VI University of Health Sciences (UM6SS), Casablanca, Morocco

**Keywords:** Thérapie cellulaire, cellules stromales mésenchymateuses, substituts osseux, consolidation osseuse, *case report*, Cell therapy, stromal mesenchymal cells, bone substitutes, bone consolidation, case report

## Abstract

L'os est le tissu humain le plus transplanté. Des interventions chirurgicales pour la réparation osseuse sont nécessaires pour diverses pathologies telles que la pseudarthrose, l'ostéoporose ou bien l'ostéonécrose. Bien que les greffes osseuses autologues restent la référence en matière de régénération osseuse, elles gardent malheureusement un bon nombre d´inconvénients: la nécessité d´une deuxième intervention et la quantité limitée du tissu prélevé. Les substituts osseux synthétiques pallient à certains de ces inconvénients mais leurs propriétés ostéo-inductives ne permettent pas de traiter des pertes osseuses importantes. Les thérapies cellulaires basées sur les cellules souches stromales mésenchymateuses (MSC) en association avec des substituts osseux peuvent être des alternatives à la greffe osseuse autologue. C´est dans ce contexte que nous rapportons le cas d´une patiente ayant une dysplasie congénitale traitée pour une pseudarthrose du fémur. L´association de cellules souches mésenchymateuses au substitut osseux nous a permis d´obtenir une consolidation après 6 mois.

## Abstract (en)

Bone is the most transplanted human tissue. Surgical interventions for bone repair are necessary for various pathologies such as nonunion, osteoporosis or osteonecrosis. Although autologous bone grafts remain the benchmark for bone regeneration, they unfortunately have a number of disadvantages: the need for a second intervention and the limited amount of tissue removed. Synthetic bone substitutes overcome some of these drawbacks, but their osteoinductive properties do not make it possible to treat significant bone losses. Cellular therapies based on mesenchymal stromal stem cells (MSC) in combination with bone substitutes may be alternatives to autologous bone grafting. It is in this context that we report the case of a patient with congenital dysplasia treated for non-union of the femur. The association of mesenchymal stem cells with bone substitute allowed us to obtain consolidation after 6 months.

## Introduction

La consolidation osseuse est un processus complexe en plusieurs étapes impliquant différents types de cellules, des matrices extracellulaires et des molécules de signalisation. Une perturbation de ce processus de consolidation osseuse peut entraîner un retard de la consolidation osseuse ou une pseudarthrose. Une intervention chirurgicale et des stratégies de régénération osseuse sont nécessaires pour stimuler la consolidation. Si la greffe osseuse autologue reste le gold standard en matière de réparation osseuse, les substituts osseux synthétiques et plus récemment l´adjonction de cellules mésenchymateuses à ces substituts osseux peuvent être des alternatives intéressantes. Nous rapportons le cas d´une patiente ayant une dysplasie congénitale traitée pour une pseudarthrose du fémur. L´association de cellules souches mésenchymateuses au substitut osseux nous a permis d´obtenir une consolidation complète après 6 mois.

## Patient et observation

**Figure 1:**
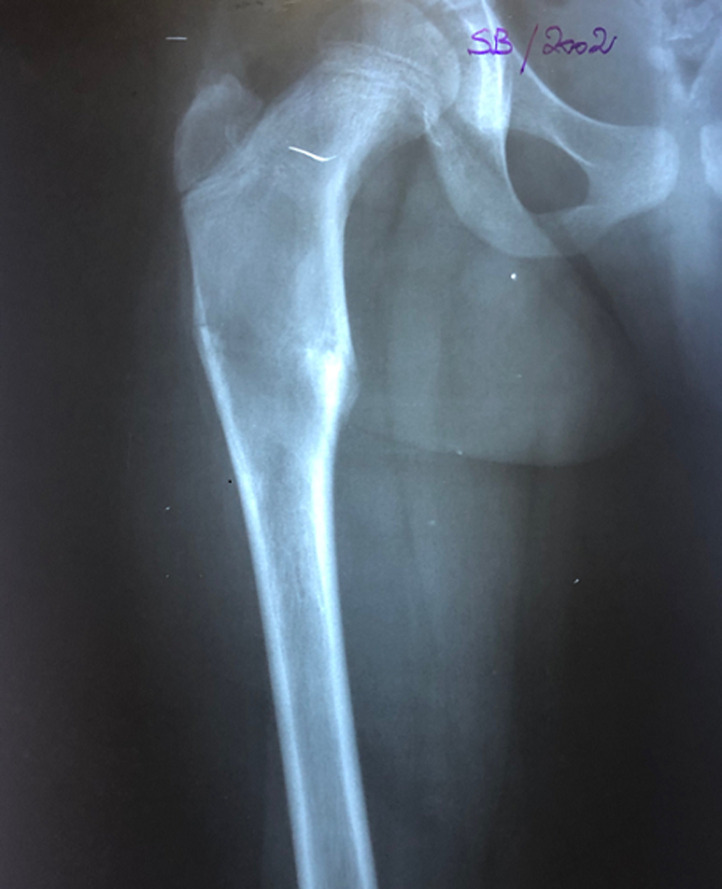
image radiographique de la lésion lytique au niveau de l´extrémité proximale du fémur dans le cadre d´une dysplasie fibreuse

**Figure 2:**
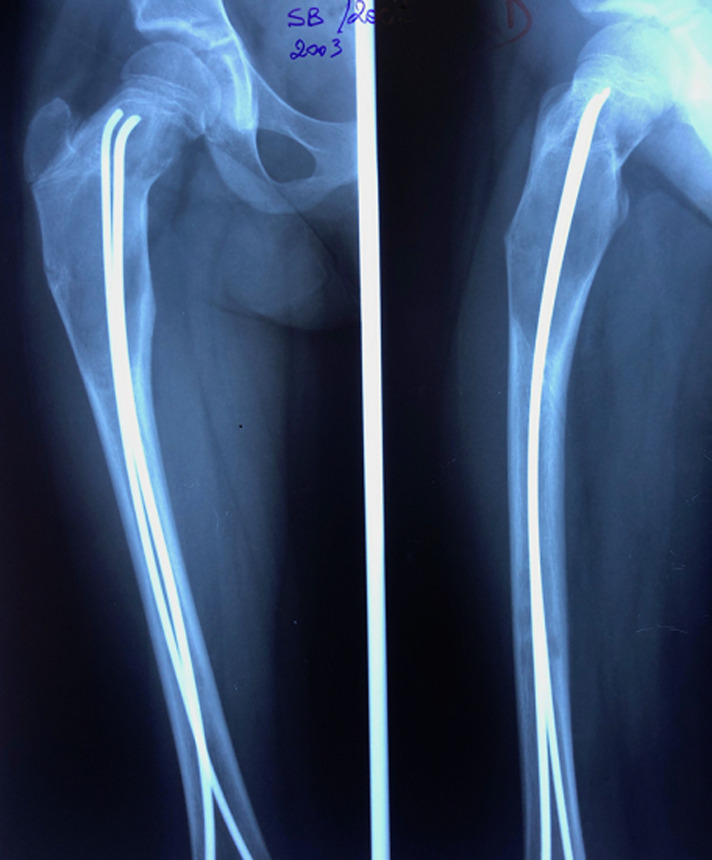
image radiographique d´un enclouage par deux broches de Metaizeau sur une lésion de dysplasie fibreuse

**Figure 3:**
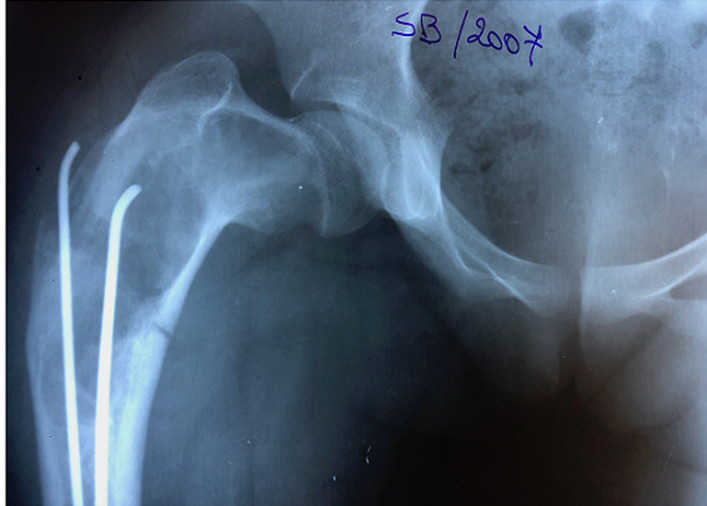
image radiographique montrant une fissure de contrainte au niveau de la partie inférieure du col fémoral

**Figure 4:**
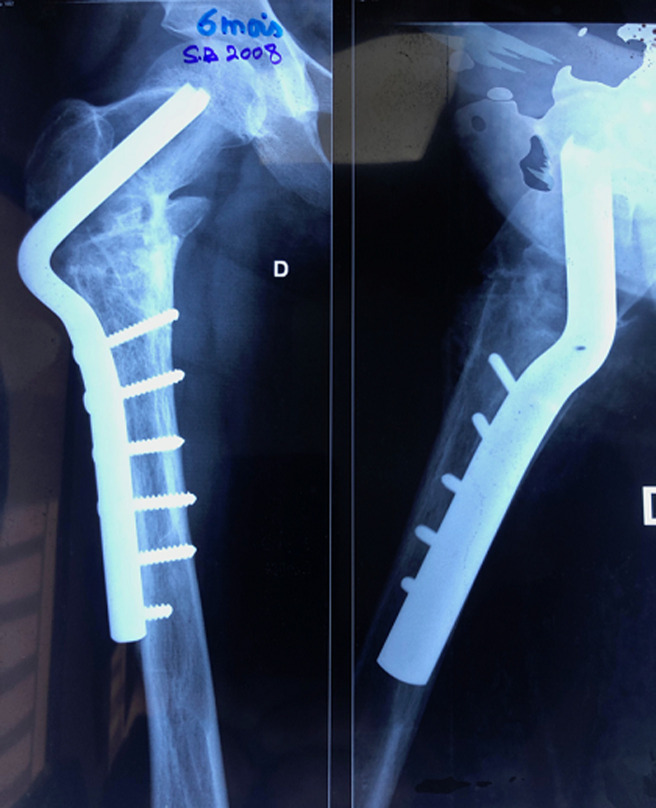
image radiographique à 6 mois de la mise en place d´une lame plaque

**Figure 5:**
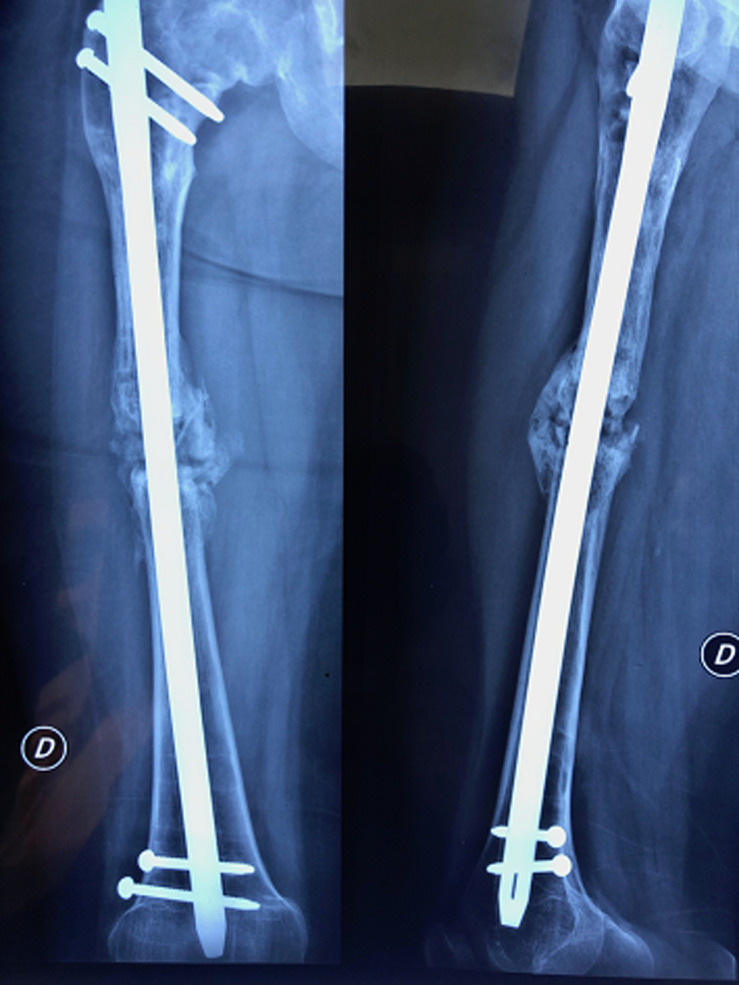
image radiographique montrant l´évolution vers la pseudarthrose après ablation de la lame plaque et la mise en place du clou fémoral verrouillé

**Figure 6:**
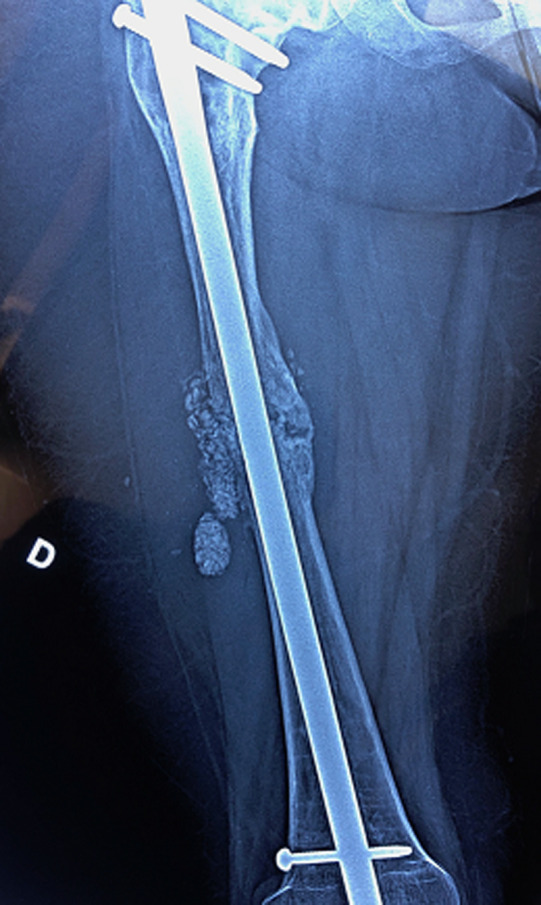
contrôle post-opératoire immédiat après traitement de la pseudarthrose par greffe de substitut osseux mélangé aux cellules mésenchymateuses stromales

**Figure 7:**
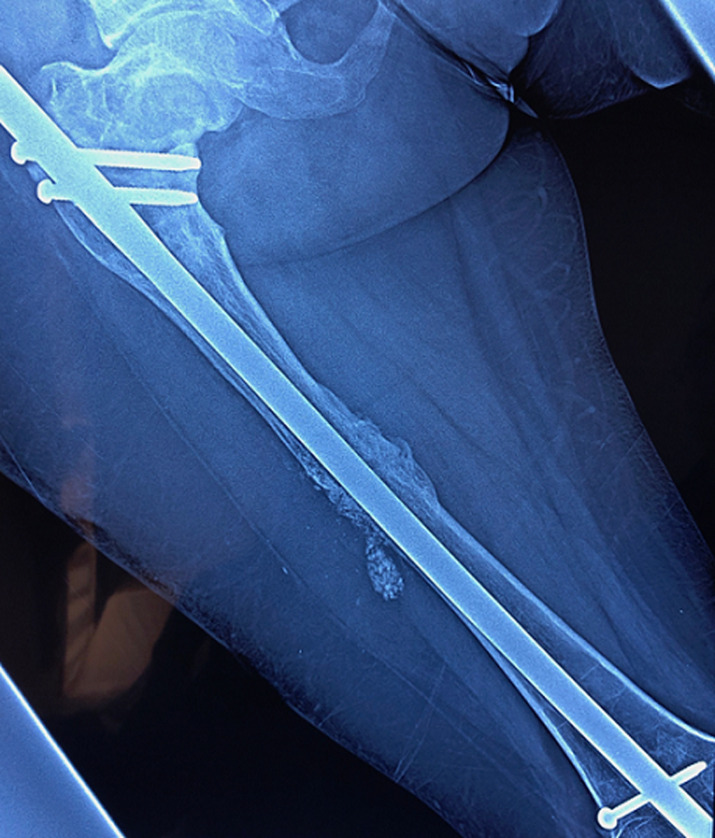
consolidation complète obtenue après 4 mois

Nous rapportons le cas d´une patiente âgée de 24 ans, diagnostiquée à l´âge de 5 ans d´un syndrome de « McCune Albright ». La patiente présentait alors une dysplasie fibreuse des os (Figure 1), des taches cutanées « café au lait » et une puberté précoce. La patiente a reçu un an après en 2001 une cure de Pamidronate (biphosphonate de deuxième génération) à raison de 180 mg tous les 6 mois, pendant 2 ans. En 2001, la patiente a présenté une fissure au niveau de l´extrémité proximale du fémur (Figure 1). Un enclouage par deux broches de Metaizeau a été réalisé (Figure 2). En 2005, la patiente a présenté une fissure de contrainte au niveau de la partie inférieure du col fémoral (Figure 3). La patiente a bénéficié de la mise en place d´une lame plaque après curetage et greffe autologue cortico-spongieuse prélevée à partir de la crête iliaque. La radio de contrôle à 6 mois (Figure 4) montre une consolidation osseuse complète. En 2015, la patiente a présenté une fracture du fémur au niveau de l´extrémité distale de la lame plaque traitée par ablation de cette lame plaque et mise en place d´un clou centromédullaire verrouillé. La fracture a malheureusement évolué vers la pseudarthrose (Figure 5). La patiente a été opérée en septembre 2018. Lors d´une première anesthésie, 50 ml de moelle osseuse étaient aspirés de la crête iliaque postérieure. Les cellules mésenchymateuses stromales étaient isolées et amplifées au laboratoire pendant 3 semaines, jusqu´à obtention de 200 millions de cellules mésenchymateuses stromales. Le jour de l´intervention, les cellules mésenchymateuses étaient mélangées à 10 cm^3^ de granules de substitut osseux synthétique pendant 1 heure, puis ce mélange a été utilisé en lieu et place d´une autogreffe spongieuse au niveau de la pseudarthrose après abord chirurgical et décortication (Figure 6). La consolidation totale a été obtenue après 4 mois (Figure 7). Il n´y a eu aucune complication clinique, biologique ou septique lié au prélèvement ou à l´utilisation des cellules mésenchymateuses.

## Discussion

L´équilibre entre la formation osseuse et la résorption osseuse permet l´entretien osseux. Lorsque cet équilibre est interrompu par une fracture osseuse, le tissu osseux possède une capacité innée de se réparer. En effet en dehors du foie, l'os est le seul tissu du corps humain à pouvoir s'auto-renouveler sans cicatrice [1]. La cicatrisation osseuse est un processus complexe en plusieurs étapes impliquant différents types de cellules, des matrices extracellulaires et des molécules de signalisation [2]. Une perturbation de la cascade de consolidation osseuse peut entraîner une pseudarthrose. Une intervention chirurgicale et des stratégies de régénération osseuse sont nécessaires pour aider à la consolidation. Les facteurs de risque des pseudarthroses des os longs comprennent le tabagisme, l'infection, l'écart de fracture postopératoire, un degré élevé de déplacement initial de la fracture et les fractures sur os pathologiques comme les dysplasies fibreuses [3]. Le Gold standard du traitement de la pseudarthrose atrophique comprend la stabilisation chirurgicale et la greffe osseuse autologue. La greffe osseuse autologue est la procédure de greffe la plus sûre et la plus efficace et implique de prélever les fragments osseux du patient sur un deuxième site chirurgical, généralement la crête iliaque, et de les greffer sur le site de la perte osseuse [4].

La greffe osseuse autologue a l´avantage de contenir des cellules osseuses pour améliorer l'ostéogenèse et des protéines pour stimuler l'ostéo-induction, tout en fournissant un échafaudage pour combler le vide osseux et pour soutenir la consolidation osseuse par ostéo-conduction. Cependant, l'autogreffe osseuse est limitée en quantité (environ 20 cm^3^) et le processus de prélèvement peut induire une morbidité, des douleurs et des complications au deuxième site chirurgical [5,6]. La procédure de récolte nécessite également une prolongation importante du temps de chirurgie pour la préparation et la collecte de tissu osseux. L'allogreffe est un autre type de greffe osseuse par lequel l'os est récupéré d'un individu pour être greffé chez un autre. Cette méthode contourne la nécessité pour le patient d'avoir des volumes suffisants d'os de bonne qualité sur le site du prélèvement. Cependant, les allogreffes ne garantissent que des propriétés ostéo-conductrices en raison des différentes étapes de traitement pour éviter le rejet immunologique. Ces allogreffes comportent par ailleurs toujours le risque de transfert de maladie, d'infection et de rejet [7,8].

Les substituts osseux sont des matériaux synthétiques biocompatibles qui pourraient être utilisés à la place de l'os transplanté pour contourner les inconvénients associés aux autogreffes et aux allogreffes. Ces biomatériaux présentent une macroporosité des espaces inter-granulaires imitant la structure de l'os trabéculaire, facilitant ainsi la perméabilité aux fluides corporels, la néo-vascularisation et la croissance osseuse. Il a également été démontré que ces biomatériaux favorisent l'adhésion, la prolifération et la différenciation ostéoblastique des cellules stromales mésenchymateuses dérivées de la moelle osseuse en ostéoblastes qui produisent la matrice de collagène qui subit ensuite une minéralisation [9]. Cependant, ces substituts osseux ne possèdent pas les propriétés ostéogéniques qui leur permettraient de réparer les gros défauts osseux. Les thérapies cellulaires basées sur la moelle osseuse ou les cellules stromales mésenchymateuses amplifiées en association avec des substituts osseux peuvent être des alternatives à la greffe osseuse autologue.

En raison de la facilité de collecte, de concentration et de réinjection dans les sites de pseudarthrose, la moelle osseuse est la thérapie cellulaire la plus couramment utilisée pour la régénération osseuse en chirurgie orthopédique. La moelle osseuse concentrée contient une fraction de cellules mononucléaires qui contribue à la fois à la vascularisation et à la cascade de la consolidation osseuse. La moelle osseuse concentrée peut également être combinée en peropératoire avec une matrice ostéo-conductrice synthétique ou naturelle (par exemple, une greffe osseuse allogénique) avant l'implantation et il a été démontré qu'elle était efficace pour réaliser la consolidation osseuse dans les pseudarthroses des os longs [10,11]. La contribution des cellules mésenchymateuses stromales à la consolidation osseuse a été bien établie dans les modèles précliniques [12,13]. En outre, plusieurs essais cliniques sont en cours pour étudier le rôle des cellules mésenchymateuses stromales dérivés de la moelle osseuse dans la consolidation des fractures des os longs, comme le résume Watson *et al*. [14]. Quarto *et al*. ont été les premiers à présenter un rapport sur les données cliniques préliminaires de trois patients, qui ont été traités avec des cellules stromales de moelle osseuse autologues et des blocs poreux d'hydroxyapatite dans les défauts osseux longs. Une fusion complète dans les trois cas a été observée [15,16]. De plus, Liebergall *et al*. ont montré que les cellules mésenchymateuses stromales de la moelle osseuse en combinaison avec une matrice osseuse déminéralisée appliquée tôt après la survenue d'une fracture du tibia distal réduisaient la durée de consolidation [17]. Notre stratégie dans la régénération osseuse des pseudarthroses des os longs implique la récolte de 30 ml de moelle osseuse du patient, le transport vers l'unité de thérapie cellulaire où les cellules mésenchymateuses stromales autologues sont isolés et étendus en culture pendant 3 semaines. Les cellules mésenchymateuses stromales sont ensuite mélangées, au niveau de la salle d´opération avec des granules de phosphate de calcium synthétiques pendant 1h pour permettre aux cellules de se fixer sur le biomatériau. Le mélange de cellules mésenchymateuses stromales autologues et de substitut osseux synthétique est finalement implanté sur le site des pseudarthroses après décortication [18].

## Conclusion

Les indications de la thérapie cellulaire sont innombrables et les promesses sont réelles dans de nombreux domaines. Les résultats encourageants des premières études cliniques suggèrent que les thérapies cellulaires pourraient constituer une thérapie d´avenir en matière de reconstruction osseuse. Les différentes techniques doivent toutefois être complémentaires aux règles de base de la prise en charge chirurgicale. Il faut faire également attention à la banalisation des pratiques, aux aspects réglementaires et aux écarts éthiques.
